# Specially Trained Matrons in Demand

**Published:** 1919-08-23

**Authors:** 


### THE MATRONS' AND SISTERS' DEPARTMENT.

### SPECIALLY TRAINED MATRONS IN DEMAND.

The nursing profession is certainly one of those in which there is plenty of room at the top. The qualities essential for success in the difficult art of directing the nursing department of institutions for the sick are not very frequently found combined in an individual who has rejected other openings for her talents and has dedicated them wholeheartedly to nursing. So many women who would make good matrons take up other careers. So many probationers marked out for distinction pass from hospital life on the conclusion of their training or soon after, in obedience to other calls. The result is that choice is restricted. In order to ensure a supply of women capable of matronship in its highest aspects, the nursing profession must study to attract the right kind of student, must give ample opportunities for additional study to such as prove their fitness and must afford them adequate prospects when they have shown their ability to rule.

It is wonderful what splendid women have adopted the career of nursing in the teeth of all the discouragements opposed to them in the past. In their entry upon the scene as probationers many of them had to bear ignominy, neglect, drudgery, [illegible] misunderstanding. It was considered unseemly for any previous experience or education to count in the smallest particular while for three or four years they were put through their paces. When they emerged as fully trained nurses at the age of twenty-six or twenty-seven, they found themselves still unfledged, barely able to command the wages of an upper housemaid, compelled to go through another long apprenticeship in quasi-subordinate work before obtaining one of those administrative posts which count as a preparation for a matronship. It is disheartening to consider how many potentially good matrons have been lost in the course of this ill-paid and uninviting apprenticeship under conditions as regards personal freedom, recreation, domestic dignity, and toil, such as might repel the most zealous. Things are a great deal better than they used to be, but changes are taking place unequally, and such circumstances as we have sketched are still likely to surround candidates who happen upon one of the backward training-schools. It is a process which eliminates the fit as surely as it encourages a soulless mediocrity.

In attempting to meet the demand for sister-tutors and matrons who have undergone a special training and are in other respects well fitted for authority, the leaders of the nursing profession have to remove many obstructions. The key-note of their policy is a higher demand on the nurse. More is expected, both in the time given to training and in zeal and concentration, but opportunity is put within her grasp. To increase the number of nurses capable of rule, it is necessary to rely first on developing nurses' powers. They are capable of so much more than has been asked of them. Latent and neglected lie talents they never dream they possess. To make them what they ought to be they must be set to higher tasks. And it is the best women who will best respond to this demand.

The number of nursing scholarships is increasing month by month. Taking its impulsion, as was fitting, from the Nightingale School where nursing ideals first found a local habitation, the movement was followed up by the College of Nursing and is permeating the Provincial training-schools. Within a short while, we venture to prophesy that nursing will be fully recognised as one of the careers for which university training is a fit preparation.

The appeal for a Working Women's College, circulated on August 7 by the Education Committee of the Y.W.C.A., is a remarkable document. The main difficulty in bringing university education within the reach of the woman worker is stated to be the fact that "she is not thought to need higher education, any more than her wealthier sister of 100 years ago was thought to need it." May we not say that the bar to higher education for the nurse was identical—she was not thought to need it? The Y.W.C.A. is often asked, we learn, "What are you going to educate your students for?" And the answer they make covers the main reasons why we demand higher education for the women who are to direct the nursing departments of our hospitals:—"We hope to educate our students for fuller and wider life in whatever sphere they may be called upon to live; except in this sense, education truly understood is not 'for' anything."

